# Genetic variations in sterol regulatory element binding protein cleavage-activating protein (*SCAP*) are associated with blood pressure in overweight/obese Chinese children

**DOI:** 10.1371/journal.pone.0177973

**Published:** 2017-05-19

**Authors:** Yi-De Yang, Jie-Yun Song, Shuo Wang, Fang-Hong Liu, Yi-Ning Zhang, Xiao-Rui Shang, Hai-Jun Wang, Jun Ma

**Affiliations:** Institute of Child and Adolescent Health, School of Public Health, Peking University, Beijing, China; Universita degli Studi di Milano, ITALY

## Abstract

**Objective:**

Previous studies demonstrated a role of variations in sterol regulatory element binding protein (*SREBP*) cleavage-activating protein (*SCAP*) in obesity and blood lipids. But the associations between *SCAP* polymorphisms and blood pressure (BP) are not clear. This study aimed to investigate the relationship between genetic variations in *SCAP* and BP phenotypes in a Chinese pediatric population.

**Methods:**

A case-control study on 702 high blood pressure (HBP) children and 1319 controls was conducted to explore the correlation between single nucleotide polymorphism markers (rs12487736 and rs12490383) of *SCAP* and BP phenotypes. The associations with continuous and categorical variables were examined by linear regression and logistic regression models under a dominant genetic model for the minor rs12487736 A allele and rs12490383 T allele.

**Results:**

The rs12487736 polymorphism was significantly associated with systolic BP (SBP) (β = 1.66, *P* = 0.003) and diastolic BP (DBP) (β = 1.35, *P* = 0.024) with age, age-squared, sex, study population and body mass index (BMI) adjusted under the dominant genetic model. The rs12490383 polymorphism was significantly associated with SBP (β = 1.71, *P* = 0.004) and SHBP (OR = 1.39, 95%CI: 1.04–1.86, *P* = 0.027). When analyzed by BMI categories, in the normal-weight children, no significant association between the *SCAP* polymorphisms and BP phenotypes was observed (all *P* > 0.05). However, in the overweight/obese children, rs12487736 was significantly associated with SBP (β = 1.6, *P* = 0.019) and SHBP (OR = 1.36, 95%CI: 1.02–1.82; *P* = 0.037), rs12490383 was associated with SBP (β = 2.04, *P* = 0.004) and SHBP (OR = 1.50, 95%CI: 1.10–2.05; *P* = 0.01).

**Conclusions:**

This study demonstrated that *SCAP* rs12487736 and rs12490383 were significantly associated with SBP and SHBP in overweight/obese Chinese children. It provided the evidence for association of *SCAP* with SBP.

## Introduction

High blood pressure (HBP) is an important risk factor for global burden of disease, it has been reported that high systolic blood pressure (SBP) accounted for 10.4 million deaths and 208.1 million disability-adjusted life-years in 2013[1]. In recent years, pediatric HBP among Chinese children is also alarming and drawing the immense attention of public health researchers[2]. Pediatric HBP can track into adulthood[3], leading to a series of cardiovascular diseases and end-organ damage[4].

It is estimated that the heritability of blood pressure (BP) is about 30%-60%[5]. However, based on a genome-wide association study of SBP and diastolic blood pressure (DBP), it is estimated that 116 independent genetic variants explain no more than 2.2% of the phenotypic variance for SBP or DBP[6]. So it is necessary and important to improve the understanding of the genetic architecture of SBP and DBP.

Sterol regulatory element binding protein (SREBP) cleavage-activating protein (SCAP) is an important regulator of cholesterol homeostasis. Variants in *SCAP* were found to affect the risk of obesity and level of serum lipids[7–9]. The rs12487736 polymorphism is a common polymorphism located in exon 16. It is associated with obesity and might contribute to the serum levels of cholesterol in different populations[7,8]. The rs12490383 polymorphism was reported to be associated with body mass index (BMI) change[9]. Recently, it was found that two polymorphisms (rs12487736 and rs12490383) in *SCAP* were related to obesity in Chinese children[10].

Mounting evidence shows that obesity and HBP are closely related[11,12], and may share some genetic background. Therefore, this study evaluated the associations between the two *SCAP* polymorphisms and BP phenotypes, including SBP, DBP, HBP, systolic HBP (SHBP) and diastolic HBP (DHBP). Furthermore, the influence of overweight/obesity on the associations was analyzed.

## Methods

### Study population

An association study was conducted in 2030 subjects from two independent study groups who were recruited from the urban region of Beijing, China. The first study group came from the study on adolescent lipids, insulin resistance, and candidate genes (ALIR). The second study group was from the Comprehensive Prevention project for Overweight and Obese Adolescents (CPOOA). The ALIR study included 937 adolescents aged 14–17 years recruited from 9 middle schools in Dongcheng District of Beijing. The CPOOA study included 1093 children and adolescents aged 7–18 years from 3 elementary and 2 middle schools in Haidian District of Beijing. The details of cluster sampling and ascertainment strategies of the two study groups have been described previously[13]. In the two studies, the children who were underweight according to the Chinese national screening criteria for malnutrition of children aged 6–18 years were excluded[14].

According to the Chinese BMI percentile criteria for screening overweight and obesity in children aged 7–18 years, the participants with age- and gender-specific BMI ≥ 95th percentile were defined as obese, while those with age- and gender-specific BMI between 85th and 95th percentiles were defined as overweight[15]. After exclusion of underweight children, those with age- and gender-specific BMI less than 85th percentile were considered as normal-weight children. SHBP and DHBP were defined as SBP or DBP ≥ the age- and sex-specific 95th percentile of a representative Chinese children population, respectively[16]. HBP was defined as SHBP and/or DHBP. We calculated Homeostasis model assessment of insulin resistance (HOMAIR) by using the HOMA Calculator version 2.2 available from the Oxford Centre for Diabetes, Endocrinology and Metabolism (www.dtu.ox.ac.uk)[17]. The quantitative insulin sensitivity check index (QUICKI) was calculated according to the formula: QUICKI = 1/log (fasting insulin (μU/ml)) + log (fasting glucose (mg/dl))[17].

The two studies were approved by the ethic committee of Peking University Health Science Center. Written informed consent was provided by all participants and, in the case of minors, their parents.

### Measurements

Anthropometric measurements, including height, weight, SBP and DBP were performed according to the standard protocols. Height and weight were measured twice, and the mean value for each participant was recorded. BP was calculated by averaging three measurements at a single visit. It was measured three times with 5-minutes time interval. BP was measured according to the recommendation of the National High Blood Pressure Education Program Working Group for Children and Adolescents[18], using an auscultation mercury sphygmomanometer with an appropriate cuff size for children. The cuff bladder width could cover 50–75% of the circumference of the arm. BP measurements were taken at least 5 minutes after resting. SBP was defined as the onset of “tapping” Korotkoff sound (K1), and DBP was defined as the fifth Korotkoff sound (K5). Of the 2030 subjects, 9 (0.4%) individuals with BP values missing were excluded.

### SNP selection and genotyping

In the present study, two polymorphisms (rs12487736[19] and rs12490383[9]) of *SCAP* were selected based on previous studies. SNPs of *SCAP* that were reported to be associated with BP and BP-related phenotypes were searched by reviewing the previous literatures. With the assumed OR of 1.25 for the risk of HBP and allele frequency not less than 0.21, the power for detecting significant association was higher than 0.80. Therefore, SNPs with minor allele frequency (MAF) ≥ 0.21 in the Chinese population were selected by using the HapMap database (http://hapmap.ncbi.nlm.nih.gov/). Additionally, our study group found that the two polymorphisms were associated with obesity[10].

Genomic DNA was isolated from blood leukocytes by the pheno-chloroform extraction method. The polymorphisms were genotyped using iPLEX assays (Sequenom, San Diego, CA, USA), which were designed with the Assay Design Suite 1.0 online software. The assay details of rs12487736 and rs12490383 are available from the authors upon request. A multiplex polymerase chain reaction was performed, and unincorporated double stranded nucleotide triphosphate bases were dephosphorylated with shrimp alkaline phosphatase followed by primer extension. The purified primer extension reaction product was spotted on to a 384-element silicon chip (SpectroCHIP, Sequenom) and analyzed in the Matrix assisted laser desorption ionization time of flight mass spectrometry (MALDI-TOF MS systems, Sequenom). The genotyping results were processed with the Typer 4.0 software (Sequenom). All the experiments were performed by investigators who were blind to the phenotypes. The genotyping call rates of rs12487736 and rs12490383 was 99.3% and 99.6%, respectively.

### Statistical analyses

The Hardy-Weinberg equilibrium was tested with the chi-square test for the genotype data of the non-HBP group. The continuous variables were described as median (Inter-Quartile Range, IQR), and differences between groups were analyzed by the Mann-Whitney U test. Analysis of covariance (ANCOVA) was used to obtain and compare means of SBP and DBP between the three genotypes of *SCAP* polymorphisms. Means compared between groups were adjusted for multiple comparisons with the least significant difference method in ANCOVA model. Age, age-squared, sex, study population and BMI were used as covariates in the ANCOVA model. Linear regression model was used to analyze the associations between the *SCAP* polymorphisms and SBP or DBP, and the regression coefficients (β) were presented. Logistic regression model was used to analyze the associations between *SCAP* polymorphisms and risk of HBP, SHBP or DHBP by calculating the odds ratio (OR) with 95% confidence intervals (95%CIs). All analyses were performed under a dominant genetic model. All P values were two-sided. SPSS for Windows (version 18.0, SPSS Inc., Chicago, IL, USA) were used for the statistical analysis. Bonferroni correction was used for adjustment of multiple testing.

Since the effect size of rs12487736 and rs12490383 on the risk of HBP or hypertension was not available, with an assumed OR of 1.25 for the risk of HBP, the statistical power to detect significant association was estimated to be greater than 90%, based on the current sample size, the corresponding MAF and the significance level of 0.05 of two-sided under dominant model. Power calculation was performed by using Quanto software (University of Southern California, Los Angeles, CA, USA).

## Results

### General characteristics

**Fig 1:**
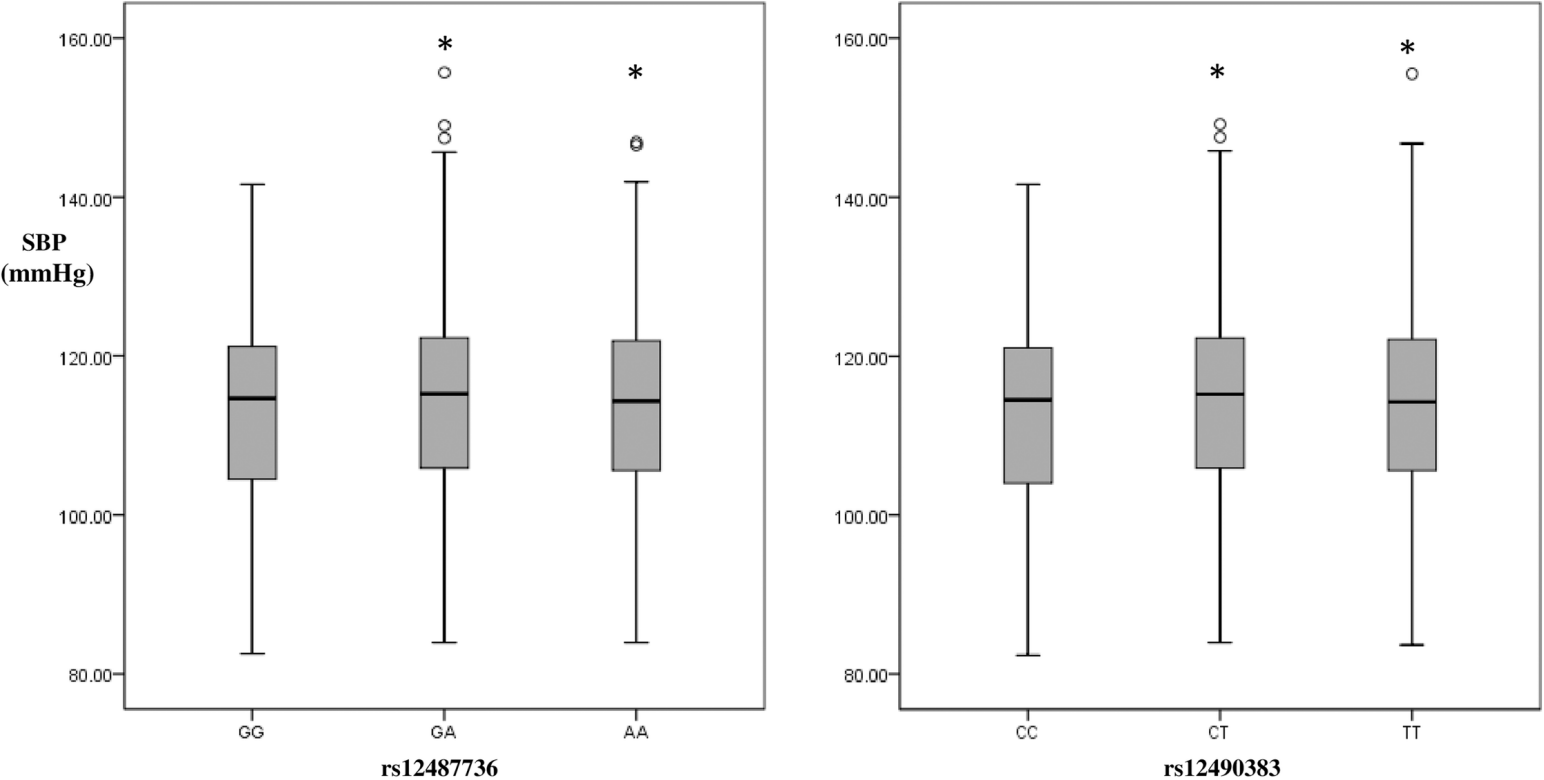
Systolic blood pressure distribution in different rs12487736/rs12490383 genotypes under ANCOVA model in Chinese children. SBP: systolic blood pressure. SBP was estimated under ANCOVA model with age, age-squared, sex, study population and BMI adjusted. *The mean of SBP were significantly different from group of genotype = GG/CC.

**Fig 2:**
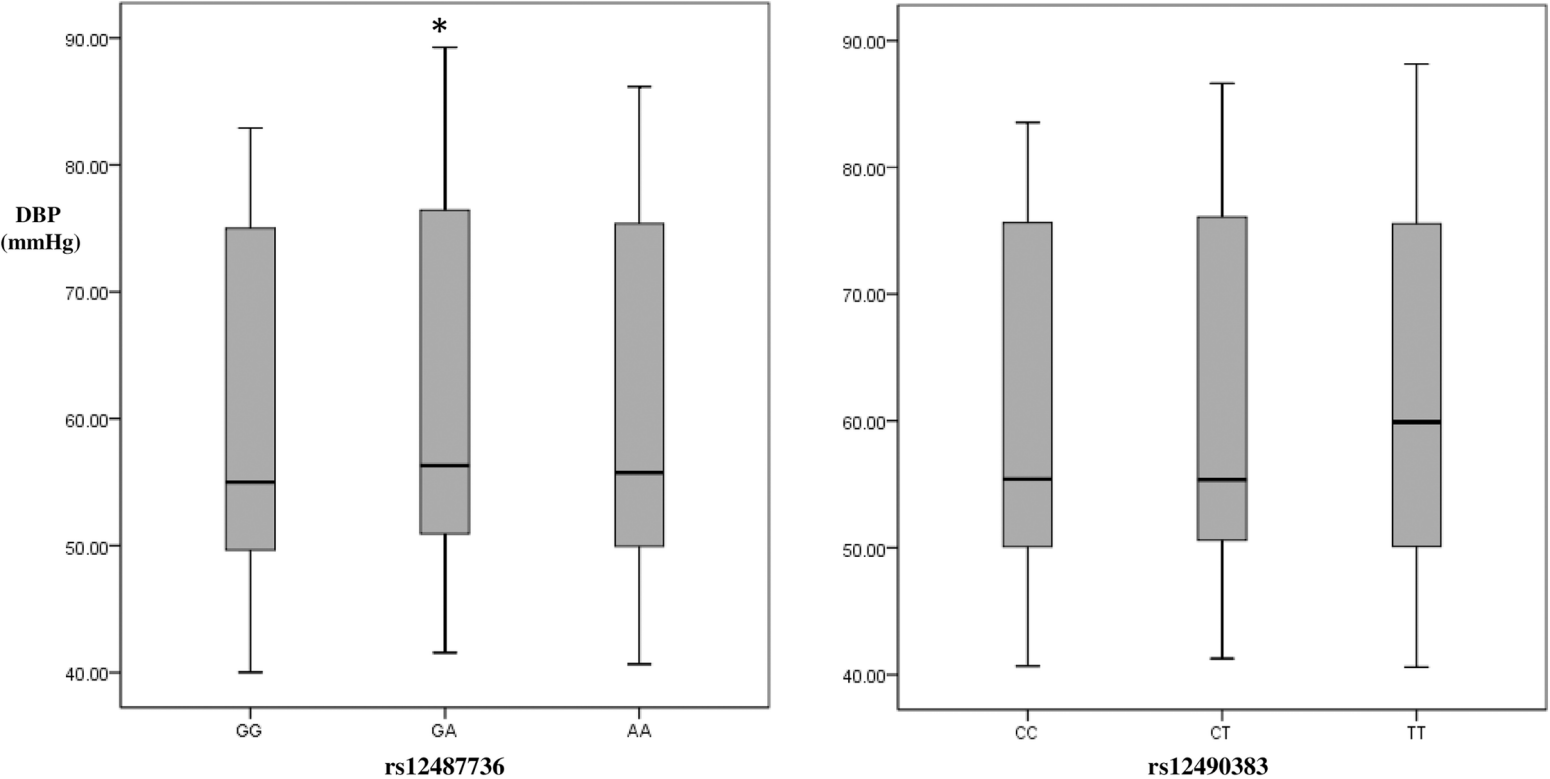
Diastolic blood pressure distribution in different rs12487736/rs12490383 genotypes under ANCOVA model in Chinese children. DBP: diastolic blood pressure. DBP was estimated under ANCOVA model with age, age-squared, sex, study population and BMI adjusted. *The mean of DBP was significantly different from group of genotype = GG/CC.

A total of 1319 non-HBP children and 702 HBP children with completed phenotype data were included in the study. The standard of exclusion of underweight participants was set before the recruitment of participants, actually there is no underweight children involved in the study. The general characteristics of the study population are shown in Table 1, with the characteristics of two independent study populations in S1 Table. The proportion of boys in the HBP group was significantly higher than that in non-HBP group (69.9% vs 54.9%, *P* < 0.001). The average age, BMI, SBP and DBP of HBP children were significantly higher than those of the non-HBP controls (all *P* < 0.001). Figs 1 and 2 showed the adjusted blood pressure distribution in different rs12487736/ rs12490383 genotypes groups with age, age-squared, sex, study population and BMI adjusted under ANCOVA model. We compared the adjusted BP levels of different genotypes carriers by using ANCOVA analysis, and found the BP levels of heterozygous and homozygotes of risk alleles were similar compared to BP levels of the wild types (Figs 1 and 2). So we used the dominant genetic model (for rs124877736, GG = 0, GA/AA = 1; for rs12490383, CC = 0, CT/TT = 1) in the present study.

**Table 1:** General characteristics of study population.

Variables	Non-HBP group	HBP group	*P-*value
n	1319	702	
Male (n(%))	724(54.9)	491(69.9)	<0.001
Age (years)	12.17±2.89	14.26±1.42	<0.001
BMI (kg/m^2^)	22.07(18.87,24.85)	26.7(24.73,29.44)	<0.001
SBP (mmHg)	106(96,112)	130(120,140)	<0.001
DBP (mmHg)	58(46,70)	80(80,80)	<0.001

Abbreviations: N, number. BMI: body mass index. SBP/ DBP/ HBP: systolic/diastolic/high blood pressure. BMI/SBP/DBP values were described as median (Inter-Quartile Range).

### Association between *SCAP* polymorphisms and BP

The two polymorphisms were in Hardy-Weinberg equilibrium in the control group (for rs12487736, *P* = 0.470, for rs12490383, *P* = 0.745). The frequency of A-allele and G-allele of rs12487736 was 47.6% and 52.4%, respectively. The frequency of T-allele and C-allele of rs12490383 was 51.7% and 48.3%, respectively. The rs12487736 and rs12490383 of *SCAP* were in strong linkage disequilibrium (*r*^2^ = 0.82). Therefore, the association between rs12487736 and BP phenotypes was similar to the association between rs12490383 and BP phenotypes. The rs12487736 polymorphism was found to be significantly associated with the SBP level (β = 1.66, 95%CI: 0.55–2.78, *P* = 0.003) and DBP (β = 1.35, 95%CI: 0.18–2.53, *P* = 0.024) under the dominant genetic model adjusted for age, age-squared, sex, study population and BMI (Table 2). The rs12490383 polymorphism was significantly associated with the SBP level (β = 1.71, 95%CI: 0.54–2.88, *P* = 0.004) (Table 2). In addition, rs12490383 was found to be associated with the risk of SHBP (OR = 1.39, 95%CI: 1.04–1.86, *P* = 0.027) (Table 3). No significant effect of the *SCAP* polymorphisms on DBP, HBP or DHBP (all *P* > 0.05) was detected. Furthermore, the associations between rs12487736/rs12490383 and SBP were still significant after Bonferroni correction for multiple testing (*P*< 0.005, 0.05 divided by 2 SNPs and 5 phenotypes).

**Table 2:** Association of SCAP polymorphisms with blood pressure level in Chinese children.

Phenotypes	rs12487736			rs12490383		
	(0 = GG, 1 = GA/AA)			(0 = CC, 1 = CT/TT)		
	β	95%CI	*P-*value*	β	95%CI	*P-*value*
SBP(mmHg)	1.66	0.55,2.78	**0.003**	1.71	0.54,2.88	**0.004**
DBP(mmHg)	1.35	0.18,2.53	**0.024**	0.45	-0.79,1.68	0.481

Abbreviations: SBP/ DBP: systolic/diastolic blood pressure. β with 95% confidence interval (CI) and *P*-value was estimated with linear regression analysis under dominant model with age, age-squared, sex, study population and BMI adjusted.

* The associations remained significant after further adjustment for lipid parameters (triglycerides /total cholesterol/low-density lipoprotein cholesterol / high-density lipoprotein cholesterol) in the multivariable models.

**Table 3:** Association of *SCAP* polymorphisms with high blood pressure phenotype in Chinese children.

Phenotypes	rs12487736			rs12490383		
	(0 = GG, 1 = GA/AA)			(0 = CC, 1 = CT/TT)		
	Β	95%CI	*P-*value*	β	95%CI	*P-*value*
SBP(mmHg)	1.66	0.55,2.78	**0.003**	1.71	0.54,2.88	**0.004**
DBP(mmHg)	1.35	0.18,2.53	**0.024**	0.45	-0.79,1.68	0.481

Abbreviations: OR: odds ratio. HBP: high blood pressure. SHBP/ DHBP: systolic/diastolic high blood pressure. OR with 95% confidence interval (CI) and *P*-value was estimated with logistic regression analysis under dominant model with age, age-squared, sex, study population and BMI adjusted.

We analyzed the association between the SCAP polymorphisms and blood lipid parameters. Carriers of the minor allele(GA/AA) of the rs12487736 showed lower high-density lipoprotein cholesterol concentration than those GG genotype carriers (β = -0.031, SE = 0.01, *P* = 0.016) under dominant model with age, age-squared, sex, study population and BMI adjusted (S2 Table). We further adjusted for blood lipid parameters (triglycerides / total cholesterol / low-density lipoprotein cholesterol / high-density lipoprotein cholesterol) in the linear regression models to analyze the associations between *SCAP* polymorphisms and BP. The associations of both polymorphisms with SBP remained significant (data not shown).

In addition, we analyzed the associations of two insulin sensitivity indices (HOMAIR / QUICKI) with rs12487736 and rs12490383. The associations were not statistically significant (*P*>0.05, S2 Table).

### Association between *SCAP* polymorphisms and BP stratified by BMI categories

The associations between the *SCAP* polymorphisms and BP phenotypes were further analyzed in different groups stratified by the BMI categories. The rs12487736 polymorphism was significantly associated with SBP (β = 1.6, SE = 0.68, *P* = 0.019) in the overweight/obese children, but not in the normal-weight children (β = 1.84, SE = 1.01, *P* = 0.069, Table 4). The rs12490383 polymorphism was significantly associated with SBP in the overweight/obese children (β = 2.04, SE = 0.72, *P* = 0.004) (all *P* > 0.05, Table 4).

**Table 4:** Association between SCAP polymorphisms and blood pressure stratified by BMI categories in Chinese children.

SNP	phenotype	BMI categories	Genotype	BP(mmHg)	β	SE	*P*-value
				Median(IQR)			
rs12487736 (0 = GG, 1 = GA/AA)	SBP	Normal-weight group	GG	102(92,112)	1.84	1.01	0.069
			GA/AA	104(94,112)			
		Overweight/obese group	GG	118(108,126)	1.6	0.68	**0.019**
			GA/AA	120(106,130)			
	DBP	Normal-weight group	GG	54(44,64)	2.19	1.22	0.073
			GA/AA	56(44,66)			
		Overweight/obese group	GG	70(54,80)	1.01	0.68	0.136
			GA/AA	70(54,80)			
rs12490383 (0 = CC, 1 = CT/TT)	SBP	Normal-weight group	CC	102(92,112)	1.03	1.06	0.336
			CT/TT	104(94,112)			
		Overweight/obese group	CC	120(108,126)	2.04	0.72	**0.004**
			CT/TT	120(106,130)			
	DBP	Normal-weight group	CC	56(44,64.5)	0.42	1.29	0.745
			CT/TT	56(44,66)			
		Overweight/obese group	CC	70(54,80)	0.46	0.71	0.518
			CT/TT	70(54,80)			

Abbreviations: IQR: Inter-Quartile Range. SE: Standard error. BMI: body mass index. SBP/DBP: systolic/diastolic blood pressure. Effect size(β) and *P*-value were estimated with linear regression analysis under dominant model with age, age-squared, sex, study population and BMI adjusted.

After adjusting for age, age-squared, sex, study population and BMI, rs12487736 was significantly associated with the risk of SHBP, with OR of 1.36 (95%CI: 1.02–1.82; *P* = 0.037) in the overweight/obese group. The rs12490383 was also significantly associated with a higher risk of SHBP (OR = 1.50, 95%CI: 1.10–2.05; *P* = 0.01) in the overweight/obese group. However, in the normal-weight group, the association between the two SCAP polymorphisms and SHBP was not significant (all *P* > 0.05) (Table 5).

**Table 5:** Association between *SCAP* polymorphisms and systolic high blood pressure risk stratified by BMI categories in Chinese children.

SNP	BMI categories	Genotype	Frequency (%)		OR(95%CI)	*P*-value
			Non-SHBP	SHBP		
rs12487736 (0 = GG, 1 = GA/AA)	Normal-weight group	GG	148(94.27)	9(5.73)	0.86(0.37,1.91)	0.678
		GA/AA	424(95.5)	20(4.5)		
	Overweight/obese group	GG	285(71.79)	112(28.21)	1.36(1.02,1.82)	**0.037**
		GA/AA	679(67.29)	330(32.71)		
rs12490383 (0 = CC, 1 = CT/TT)	Normal-weight group	CC	124(93.94)	8(6.06)	0.76(0.32,1.78)	0.527
		CT/TT	450(95.54)	21(4.46)		
	Overweight/obese group	CC	246(72.78)	92(27.22)	1.50(1.10,2.05)	**0.01**
		CT/TT	719(67.13)	352(32.87)		

Abbreviations: SHBP: systolic high blood pressure. Odds ratio(OR) with 95% confidence interval (CI) and *P*-value was estimated with logistic regression analysis under dominant model with age, age-squared, sex, study population and BMI adjusted.

The rs12487736 and rs12490383 were not associated with DBP, neither in the normal-weight group nor in the overweight/obese group (all *P* > 0.05) (Table 4). The stratified associations between the two SCAP polymorphisms and the risk of HBP/DHBP were tested. No significant associations were detected, except the association of rs12487736 with the risk of HBP in the overweight/obese children (OR = 1.34, 95%CI: 1.01–1.78, *P* = 0.041, S3 and S4 Tables).

## Discussion

This was the first study to elucidate the association of common genetic variants (rs12487736 and rs12490383) in *SCAP* with BP phenotypes in a pediatric population. The findings showed both rs12487736 and rs12490383 were associated with SBP and SHBP. The stratified analyses revealed that only in the overweight/obese children rs12490383 were associated with SBP and SHBP, and rs12487736 was associated with SBP/HBP/SHBP.

A review of genome-wide association study (GWAS) on BP did not support *SCAP* as a candidate gene for BP. While the present results showed that two polymorphisms of *SCAP* were associated with BP. The discrepancy may be due to the difference in age, ethnicity or BMI distribution. The results also showed that *SCAP* rs12487736 genotype GA/AA and rs12490383 genotype CT/TT were associated with a higher SBP level and a higher risk of SHBP. No previous study directly evaluated the association between the *SCAP* polymorphisms and BP phenotypes, but one study indicated the same direction. Friedlander et al[20] demonstrated different prevalence of hypertension in different rs12487736 genotypes carriers (GA/GG carriers: 20.2%; AA carriers: 29.9%) in 340 American female adults. Since insulin resistance is closely related with hypertension, especially for obesity hypertension [21], we further examined the association between HOMAIR/QUICKI with *SCAP* polymorphisms, and we found the *SCAP* polymorphisms were not associated with insulin sensitivity.

The rs12487736 polymorphism is an exonic missense mutation located at an exon (A to G transition, 2386 A>G) of human *SCAP* gene and it leads to amino acid change of isoleucine-to-valine substitution (I796V) [22]. Future functional studies are still needed to explore the underlying mechanism between SCAP and SBP. In our population MAF of the polymorphisms are relatively high (MAF = 0.476 / 0.483 for rs12487736 and rs12490383), suggesting strong conservative value of the two polymorphisms across evolution. We found the rs12487736 polymorphism was in strong linkage disequilibrium with rs12490383 (*r*^*2*^ = 0.82) in the present study, while the two variants were just in weak linkage disequilibrium in Caucasian (*r*^*2*^ = 0.223), which suggested ethic difference. Since we did not collect data about the effect of this polymorphism on SCAP activity, we could only provide associative information. Future deep functional study should be warranted to explore the causal effects of variants on SCAP activity and their further effect on BP.

In the analyses stratified by BMI categories, the association between SBP/SHBP and rs12487736 was found to be statistically significant in the overweight/obese subjects. In the normal-weight subjects, the association between the two *SCAP* polymorphisms and SBP/SHBP did not reach statistical significance, which might due to the difference in its effect between the two groups. Therefore, the overweight/obese children carrying rs12487736 genotype GA/AA should be a high risk population for controlling SHBP. Since obesity is associated with rs12487736 and SBP, the stratified analysis indicated that the obesity status was likely to be a confounding factor influencing this association between SBP and rs12487736.

SCAP combines with SREBPs through its carboxy-terminal domain to forge SCAP-SREBP complex. Working with insulin induced gene proteins (INSIGs), the complex transfers SREBPs from the endoplasmic reticulum to the Golgi apparatus under feedback regulation of cholesterol levels, finally affecting the synthesis of lipid[23]. Several studies showed that variations in *SCAP* gene might play a role in the lipid level[19,22] and progression of atherosclerosis[24,25]. Dyslipidemia, as an important predictor of cardiovascular disease (CVD), is also related to hypertension. Both genetic study[26] and prospective studies[27,28] demonstrated that an abnormal lipid profile was associated with the development of future hypertension. Halperin et al. reported that men with the highest quintile(80–100%) of total cholesterol (TC), non-high density lipoprotein-cholesterol (non-HDL-C) and TC/HDL-C ratio had increased risks of hypertension by 23%, 39% and 54%, respectively, compared with subjects in the lowest quintile(0–20%)[27]. We further analyzed the associations between rs12487736 and lipid parameters, and found rs12487736 GG genotype was associated with higher HDL-C. There are several previous studies evaluated rs12487736 and lipid parameters or pharmacogenomic response to cholesterol lowering drugs. A Chinese adult study showed that the variant was not associated with lipid parameters [29]. Another study found SCAP-796I isoform (A allele) is associated with higher LDL in Caucasian female[7]. A study in Chilean hypercholesterolemic individuals demonstrated that rs12487736 were not associated with pharmacogenomic response to atorvastatin[8], however, rs12487736 polymorphism G allele carriers had a significant better hypolipidemic response than AA carriers in an Indian study[30].Further adjustment for blood lipid parameters barely influenced the significant associations between *SCAP* polymorphisms and BP, which showed that the associations between *SCAP* polymorphisms and BP are independent of blood lipid level. Therefore, further studies on the association of lipid-related genes with BP would be helpful to clarify the etiology of hypertension.

Of note, the hypertension protective allele (G) of rs12487736 polymorphism was demonstrated as a risk factor for obesity (OR = 1.15, 95%CI: 1.01–1.32, P = 0.039)[10]. These findings were in contrast with the positive phenotypic link between obesity/BMI and hypertension/BP[11]. Similarly, other gene loci have been found to have the opposite effects on obesity and hypertension. The BMI-increasing allele of rs13107325 in *SLC39A8* showed a protective effect on DBP. The association of a higher BMI-GRS (genetic risk score) with decreased BP was also reported[31]. The reason for this discrepancy is that the genetic variant might be “evolutionarily” beneficial when a genetic mutation favorable to one trait can be harmful to another trait[32]. However, the underlying mechanism how *SCAP* affect both obesity and BP remains to be explored.

The strength of the present study was that it was conducted in children. Children have higher hypertension heritability and most HBP children have simple hypertension without complications. This helps to identify genetic effects on BP. The finding that *SCAP* variants affect BP only in overweight/obese children but not in normal-weight children would be helpful in developing early prevention strategy for pediatric HBP.

However, the present study had several limitations. The first was the limited sample size. The findings of the present study need further replication in other independent population groups. Secondly, only two variants of *SCAP* were studied. Other variants in *SCAP* influencing BP phenotypes might exist. Further studies should involve more variants of *SCAP*. Thirdly, as an important cardiovascular risk factor, only BP measurement was used to assess the hypertensive status, we didn’t collect data on arterial stiffness indices, such as arterial compliance and pulse wave velocity, which are also predictive of cardiovascular risk in a pediatric population[33]. And BP measurement was performed by a single-visit assessment is a limitation of our study. Moreover, only a few confounders were investigated in the present study; other factors affecting BP levels in children, such as exercise or salt consumption, were not considered.

In conclusion, this study found an association of rs12490383 polymorphism in *SCAP* with SBP and risk of SHBP, rs12487736 was associated with SBP, especially in overweight/obese children. Weight loss in the overweight children might be beneficial, since the association of rs12490383/rs12487736 with SBP/SHBP was not significant in normal weight children. The present results provided the evidence of the association of *SCAP* with SBP. Further functional studies of *SCAP* gene are needed to clarify the underlying mechanism for the effect of *SCAP* genetic variation on BP or HBP.

## Supporting information

S1 Table Characteristics of the study groups. (DOC)

S2 Table Associations between *SCAP* polymorphisms and metabolic parameters. (DOC)

S3 Table Association between *SCAP* polymorphisms and high blood pressure risk by BMI categories in Chinese children. (DOC)

S4 Table Association between *SCAP* polymorphisms and diastolic high blood pressure risk by BMI categories in Chinese children. (DOC)
