# Correction: Genetic variations in sterol regulatory element binding protein cleavage-activating protein (*SCAP*) are associated with blood pressure in overweight/obese Chinese children

**DOI:** 10.1371/journal.pone.0221612

**Published:** 2019-08-20

**Authors:** Yi-De Yang, Jie-Yun Song, Shuo Wang, Fang-Hong Liu, Yi-Ning Zhang, Xiao-Rui Shang, Hai-Jun Wang, Jun Ma

Table 3 mistakenly appears as a copy of Table 2. Please see the correct Table 3 here.

**Table 3:** Association of *SCAP* polymorphisms with high blood pressure phenotype in Chinese children.

Phenotypes	rs12487736			rs12490383		
	(0 = GG, 1 = GA/AA)			(0 = CC, 1 = CT/TT)		
	OR	95%CI	*P-*value	OR	95%CI	*P-*value
HBP	1.26	0.98, 1.64	0.076	1.18	0.90, 1.54	0.230
SHBP	1.29	0.98, 1.69	0.071	1.39	1.04, 1.86	**0.027**
DHBP	1.20	0.89, 1.61	0.244	1.07	0.78, 1.48	0.669

Abbreviations: OR: odds ratio. HBP: high blood pressure. SHBP/ DHBP: systolic/diastolic high blood pressure. OR with 95% confidence interval (CI) and *P*-value was estimated with logistic regression analysis under dominant model with age, age-squared, sex, study population and BMI adjusted
