# Supplementary material for: Genetic variations in sterol regulatory element binding protein cleavage-activating protein (SCAP) are associated with blood pressure in overweight/obese Chinese children
Source: PLoS One. 2017 May 19;12(5):e0177973. doi: 10.1371/journal.pone.0177973 (PMC5438183; doi:10.1371/journal.pone.0177973)
Supplement: S1 Table — (DOC) [file pone.0177973.s001.doc]

| **S1 Table. Characteristics of the study groups** | | | | |
| --- | --- | --- | --- | --- |
| variable | **ALIR study** | | **CPOOA study** | |
|  | non-HBP | HBP | non-HBP | HBP |
| n | 377 | 552 | 942 | 150 |
| Male (n(%)) | 214(56.8) | 390(70.7) | 510(54.1) | 101(67.3) |
| Age(years) | 14.62±0.64 | 14.65±0.55 | 11.19±2.86 | 12.83±2.39 |
| BMI(kg/m^2^) | 24.81(22.61,26.31) | 26.9(25.2,29.74) | 20.87(18.13,23.54) | 25.77(22.53,28.57) |
| SBP(mmHg) | 110(110,116) | 130(120,140) | 102(94,110) | 128(124,136) |
| DBP(mmHg) | 70(70,70) | 80(80,80) | 50(42,60) | 60(50,70) |
| Abbreviations: N, number. BMI: body mass index. SBP/ DBP/HBP: systolic/diastolic/high blood pressure. BMI/SBP/DBP were described as Median(Inter-Quartile Range). | | | | |
|  |  |  |  |  |
|  |  |  |  |  |
