# Supplementary material for: Genetic variations in sterol regulatory element binding protein cleavage-activating protein (SCAP) are associated with blood pressure in overweight/obese Chinese children
Source: PLoS One. 2017 May 19;12(5):e0177973. doi: 10.1371/journal.pone.0177973 (PMC5438183; doi:10.1371/journal.pone.0177973)
Supplement: S2 Table — (DOC) [file pone.0177973.s002.doc]

| **S2 Table. Associations between SCAP polymorphisms and metabolic parameters.** | | | | | | | | |
| --- | --- | --- | --- | --- | --- | --- | --- | --- |
| Variables | rs12487736 | | | | rs12490383 | | | |
|  | GG | GA/AA | β (SE) | *P* | CC | CT/TT | β (SE) | *P* |
| Total cholesterol | 4.26(3.79,4.73) | 4.21(3.78,4.73) | -0.031(0.04) | 0.391 | 4.28(3.82,4.71) | 4.21(3.77,4.74) | -0.039(0.04) | 0.296 |
| Triglycerides | 0.85(0.66,1.16) | 0.83(0.63,1.12) | -0.003(0.02) | 0.887 | 0.85(0.66,1.16) | 0.83(0.63,1.12) | 0.014(0.02) | 0.560 |
| Low-density lipoprotein cholesterol | 2.26(1.91,2.73) | 2.29(1.92,2.75) | 0.024(0.03) | 0.464 | 2.32(1.93,2.73) | 2.27(1.91,2.75) | 0.001(0.03) | 0.983 |
| High-density lipoprotein cholesterol | 1.22(0.84,1.54) | 1.16(0.84,1.51) | -0.031(0.01) | 0.016 | 1.23(0.84,1.54) | 1.16(0.83,1.51) | -0.023(0.01) | 0.080 |
| HOMA-IR | 1.6(1,2.6) | 1.6(0.9,2.6) | 0.032 (0.09) | 0.724 | 1.6(1,2.6) | 1.6(0.9,2.6) | -0.001(0.003) | 0.843 |
| QUICKI | 0.33(0.31,0.35) | 0.33(0.31,0.36) | 0.420 (0.96) | 0.660 | 0.33(0.31,0.36) | 0.33(0.31,0.36) | -0.002(0.003) | 0.501 |
| β and *P* were estimated with linear regression analysis under dominant model with age, age-squared, sex, study population and BMI adjusted. HOMA-IR: Homeostasis model assessment of insulin resistance. QUICKI: The quantitative insulin sensitivity check index.SE: standard error. IQR: Inter-Quartile Range. | | | | | | | | |
