# Supplementary material for: Genetic variations in sterol regulatory element binding protein cleavage-activating protein (SCAP) are associated with blood pressure in overweight/obese Chinese children
Source: PLoS One. 2017 May 19;12(5):e0177973. doi: 10.1371/journal.pone.0177973 (PMC5438183; doi:10.1371/journal.pone.0177973)
Supplement: S3 Table — (DOC) [file pone.0177973.s003.doc]

| **S3 Table. Association between SCAP polymorphisms and high blood pressure risk by BMI categories in Chinese children.** | | | | | | |
| --- | --- | --- | --- | --- | --- | --- |
| SNP | BMI categories | Genotype | Frequency (%) | | OR ^a^ | *P*-value |
|  |  |  | Non-HBP | HBP | (95%CI) |  |
| rs12487736  (0=GG, 1=GA/AA) | Normal-weight group | GG | 141(89.81) | 16(10.19) | 0.97(0.51, 1.84) | 0.919 |
|  |  | GA/AA | 397(89.41) | 47(10.59) |  |  |
|  | Overweight/obese group | GG | 230(57.93) | 167(42.07) | 1.34(1.01,1.78) | 0.041 |
|  |  | GA/AA | 542(53.72) | 467(46.28) |  |  |
| rs12490383  (0=CC, 1=CT/TT) | Normal-weight group | CC | 117(88.64) | 15(11.36) | 0.79(0.41,1.54) | 0.493 |
|  |  | CT/TT | 423(89.81) | 48(10.19) |  |  |
|  | Overweight/obese group | CC | 194(57.40) | 144(42.60) | 1.29(0.95,1.73) | 0.098 |
|  |  | CT/TT | 577(53.87) | 494(46.13) |  |  |
| Abbreviations: HBP: high blood pressure. ^a^ Odds ratio(OR) with 95% confidence interval (CI) and P-value was estimated with logistic regression analysis under dominant model with age, age-squared, sex, study population and BMI adjusted. | | | | | | |
