# Supplementary material for: Genetic variations in sterol regulatory element binding protein cleavage-activating protein (SCAP) are associated with blood pressure in overweight/obese Chinese children
Source: PLoS One. 2017 May 19;12(5):e0177973. doi: 10.1371/journal.pone.0177973 (PMC5438183; doi:10.1371/journal.pone.0177973)
Supplement: S4 Table — (DOC) [file pone.0177973.s004.doc]

| **S4 Table. Association between SCAP polymorphisms and diastolic high blood pressure risk by BMI categories in Chinese children.** | | | | | | |
| --- | --- | --- | --- | --- | --- | --- |
| SNP | BMI categories | Genotype | Frequency (%) | | OR ^a^ | *P*-value |
|  |  |  | Non-DHBP | DHBP | (95%CI) |  |
| rs12487736  (0=GG, 1=GA/AA) | Normal-weight group | GG | 149(94.90) | 8(5.10) | 1.24(0.51,2.98) | 0.64 |
|  |  | GA/AA | 411(92.57) | 33(7.43) |  |  |
|  | Overweight/obese group | GG | 266(67.00) | 131(33.00) | 1.21(0.88,1.67) | 0.243 |
|  |  | GA/AA | 643(63.73) | 366(36.27) |  |  |
| rs12490383  (0=CC, 1=CT/TT) | Normal-weight group | CC | 124(93.94) | 8(6.06) | 0.92(0.38,2.27) | 0.862 |
|  |  | CT/TT | 438(92.99) | 33(7.01) |  |  |
|  | Overweight/obese group | CC | 224(66.27) | 114(33.73) | 1.11(0.79,1.56) | 0.553 |
|  |  | CT/TT | 685(63.96) | 386(36.04) |  |  |
| Abbreviations: DHBP: diastolic high blood pressure. ^a^ Odds ratio(OR) with 95% confidence interval (CI) and P-value was estimated with logistic regression analysis under dominant model with age, age-squared, sex, study population and BMI adjusted. | | | | | | |
